# Peering into peer-review at *GigaScience*

**DOI:** 10.1186/2047-217X-2-1

**Published:** 2013-01-24

**Authors:** Scott C Edmunds

**Affiliations:** 1 GigaScience, BGI HK Ltd., 16 Dai Fu Street, Tai Po Industrial Estate, Hong Kong

## Abstract

Fostering and promoting more open and transparent science is one of the goals of *GigaScience*. One of the ways we have been doing this is by throwing light on the peer-review process and carrying out open peer-review as standard. In this editorial, we provide our rationale for undertaking this policy, give examples of our positive experiences to date, and encourage others to open up the normally opaque publication process.

## Background

One of the big scientific trends of the past year has been the high profile that open-access and more open methods of performing science has received. With the Elsevier boycott, the Finch report in the UK, and the launch of a number of innovative new schemes in publishing open-access research and data (including *Figshare*, *F1000 Research*, *eLife*, *PeerJ* and of course *GigaScience*), 2012 has been talked of as the year of an “academic spring” that has started to shake up the centuries old, stuffy and closed system of scientific discourse [1].

On top of changes to the way scientists and readers are demanding they can access and mine the literature and data, and with the new incentives and mechanisms to release and publish data, the process and deficiencies of peer-review has also come under the spotlight. Developing open source software is collaborative and interactive and so, if much of the scientific community are comfortable and find it constructive working in this manner, there is no reason why peer-review should not follow a similar process [2]. Many newly launched journals have tried to become more transparent, using systems such as post-publication peer-review (e.g. *Biology Direct* and *F1000 Research*), pre-print servers, providing access to anonymized (e.g. EMBO journals) or partial parts of the peer-review history (e.g. *eLife*), or encouraging reviewers to opt-into open peer-review (*PeerJ*, and experimented with a little at *PLOS One*).

At *GigaScience,* we have decided to take this process one step further and ask for open peer-review as default, and as our aims are to promote more open, reproducible and transparent-science, we feel it promotes accountability, fairness, and importantly gives credit to reviewers for their hard efforts. Our co-publisher BioMed Central is a pioneer in this area, and on top of the BMC Series medical journals having open peer-review since their launch in the year 2000, our stablemates and fellow data-focused journals, *BioData Mining* and *Biology Direct*, also have been following open models for a number of years as well.

## Full text

Tailoring our peer-review process to handle such data heavy articles has been a learning process, but now that sufficient examples of peer-reviewed (non-editorial) content have been published in *GigaScience*, it is now a good time to look back and highlight how it has gone so far. Last month provided an excellent example of this where we published an updated version of BGI’s popular SOAPdenovo software application, a state-of-the-art tool for *de novo* genome assembly [3].

Stating that a software application can perform better than other computational tools with the same functionality is one thing, but to justify and prove this in review, testing by independent peers is needed, and the larger and more complicated a study and its associated data is, the more challenging this can be. In order to ease, throw light and credit the reviewers in this process, *GigaScience* uses a much more transparent, accountable and open peer-review process. Tailoring the process for such data heavy studies, our criteria for publication is based more on the relative amount of data created or used, and transparency and availability more than subjective and unpredictable measures such as supposed “impact”.

## Open peer-review, *GigaScience* style

During peer review, we can host all of the supporting information and data (with several papers having datasets close to 100 GB in size so far) and our curators make all of it available to the peer-reviewers from our FTP servers. In the case of SOAPdenovo2, we worked with several groups of expert reviewers who thoroughly tested the software against various tools and datasets to ensure the claims made by the authors were correct. In this case, all 8 reviewers consented for their names to be in their reports that are now available to view from the pre-publication history section associated with our published articles (see http://www.gigasciencejournal.com/content/1/1/18/prepub).

One of the main arguments for the anonymous peer-review system is that “anonymity creates a safe place”, and more junior researchers may be reluctant to be critical of more senior authorities in their field for fear of comeback or to curry favour [4]. To counter this issue, reviewers for *GigaScience* have the ability to provide confidential comments to the editors (particularly on ethical and policy issues), as well as have the option to opt-out and have their name removed from reports if they have reasons to remain anonymous.

Despite offering this opt-out, it is encouraging that none have asked to do this for all of the papers we have reviewed so far. The reports from our reviewers have generally been very constructive, and previous studies on open peer-review have also found that quality and courteousness of reviews were increased [5], with little, if any, negative effects [6]. By making the process more open and transparent, competing interests and biases are reduced, and reviewers are able to take credit for the hard efforts they have put into the review process, and even declare and include it in their CV if they so wish. The benefits of this increased transparency to readers are also useful, as they do not have to take it on trust that published manuscripts were reviewed by qualified reviewers, and for educational purposes, they can see good examples of how peer review operates. This increased transparency has already boosted the profile, reproducibility and utility of the SOAPdenovo2 study, with groups using all of the detailed reviews, source code and supplementary materials to carry out post-publication assessment and review of the study, producing blogs and a wiki parsing and community annotating the 40,000 lines of code in the application [7].

## Promoting reproducibility, *GigaScience* style

On top of improving transparency and reproducibility during the peer-review of data-heavy studies, *GigaScience* also carries this over to the publication process, of which this paper is also an excellent example. In addition to SOAPdenovo2 meeting our requirements of being open source and having its code hosted in a repository, the authors provide detailed pipelines with the tools including the commands and necessary utilities to reproduce the different tests carried out in the paper. The SOAPdenovo2 paper is associated with 78 GB of test data, tools and scripts, which is much larger than other journals are currently able to handle, and these have been made available from our http://gigadb.org database as separate citable DOIs.

We feel that it is important to credit methodology as well as data production, and whilst we have previously published data packages combining reference datasets and tools before, SOAPdenovo2 is the first paper that we have given separate DOIs to the tools [8] and data [9]. The logic for doing this is that both can now be credited to potentially different groups of authors, and the data and analyses may be used and cited independently of each other, and each can be tracked and credited to each author via DOIs.

As this process is still evolving and being fine-tuned, we would welcome any feedback. Many journals are tentatively starting to experiment going down a partially more open route, but from our positive experiences so far we would encourage them and others to be bold and embrace full transparency.

## Competing interests

The author is employed by *GigaScience* and BGI Hong Kong.

## Authors’ contributions

SCE wrote the editorial based on the editorial policies of *GigaScience.*
